# Emerging Roles of Viroporins Encoded by DNA Viruses: Novel Targets for Antivirals?

**DOI:** 10.3390/v7102880

**Published:** 2015-10-16

**Authors:** Jamie Royle, Samuel John Dobson, Marietta Müller, Andrew Macdonald

**Affiliations:** School of Molecular and Cellular Biology, Faculty of Biological Sciences and Astbury Centre for Structural Molecular Biology, University of Leeds, Leeds LS2 9JT, UK; bs12jr@leeds.ac.uk (J.R.); bs11s3jd@leeds.ac.uk (S.J.D.); M.Muller@leeds.ac.uk (M.M.)

**Keywords:** DNA virus, viroporin, papillomavirus, polyomavirus

## Abstract

Studies have highlighted the essential nature of a group of small, highly hydrophobic, membrane embedded, channel-forming proteins in the life cycles of a growing number of RNA viruses. These viroporins mediate the flow of ions and a range of solutes across cellular membranes and are necessary for manipulating a myriad of host processes. As such they contribute to all stages of the virus life cycle. Recent discoveries have identified proteins encoded by the small DNA tumor viruses that display a number of viroporin like properties. This review article summarizes the recent developments in our understanding of these novel viroporins; describes their roles in the virus life cycles and in pathogenesis and speculates on their potential as targets for anti-viral therapeutic intervention.

## 1. Introduction

As obligate intracellular parasites, viruses have evolved a myriad of strategies to manipulate the host cell environment to one that is conducive for virus replication. Research over recent decades has identified a group of virus-encoded proteins able to mediate the passage of ions and solutes across cellular membranes, termed viroporins [1,2]. The majority of viroporins described are small (less than 120 amino acids) and contain one or two transmembrane domains (TMD), although a small number of larger viroporins have been shown to encode up to three putative TMD [3]. Whilst high-resolution structural information is currently only available for a limited number of viroporins [4,5,6,7,8], a bio-informatic approach has often been successfully employed to identify key features in viroporins that lack any structural information [1,3]. Their small size necessitates that viroporins must oligomerize in membranes to form an active channel complex. Formation of these high-order complexes is often observed in mild detergents such as 1,2-diheptanoyl-*sn*-glycero-3-phosphocholine (DHPC) [3,9] and is likely to be mediated by hydrophobic interactions between the TMD of each monomer; although in some viroporins basic residues adjacent to the TMD may facilitate membrane binding and insertion [10,11]. Known viroporins have been placed into distinctive classes based on the number of TMD and the orientation of their carboxyl termini relative to the endoplasmic reticulum (ER) membrane [2]. Undoubtedly, this classification system is useful for cataloguing the expanding number of viroporins, however, it will need to adapt in order to accommodate those few viroporins encoding three TMD and it will also need to take into account the dynamic nature of membrane proteins in lipids, which are capable of altering the orientation of their termini depending on the lipid environment [3,12].

Modulation of ionic homeostasis within specific cellular compartments allows for viroporins to manipulate a wide range of cellular processes from autophagy [13,14,15], trafficking [16,17], inflammation [18,19], transformation [3] to cell survival [20]. Due to these broad perturbations to host cell physiology, it is not surprising that viroporin function has been shown to assist in all stages of the virus life cycle including entry, membrane penetration, genome replication and virus egress [1,2].

The existence of virus encoded pore-forming proteins was initially postulated nearly four decades ago [21]. However, it was observations that the M2 protein of Influenza A virus (IAV) was able to form a tetramer [22] and raise intracellular pH [23,24] that provided the first clues to its role as an ion channel. Pioneering studies in *Xenopus* oocytes demonstrated M2-dependent currents that could be blocked by addition of the anti-viral compound Amantadine [25]. Following this, viroporins were swiftly identified from a number of RNA virus families. Whilst IAV M2 remains the paradigm, viroporins have now been described in a number of virus families including the *Flaviviridae*, *Picornaviridae*, *Retroviridae*, *Coronaviridae*, *Reoviridae* and *Paramyxoviridae* [1,26,27,28,29,30,31,32]. To date, the overwhelming majority of viroporins have been identified in RNA viruses. However, given the myriad of cellular processes that they are able to modulate, it is logical to assume that all viruses might benefit from encoding such a protein. Recent findings have now identified proteins that exhibit a number of viroporin characteristics encoded by members of the *Polyomaviridae* and *Papillomaviridae*. This review will summarize our understanding of these putative viroporins, describe their known functions and attempt to highlight how possible ion channel activity may aid the life cycles of these small DNA tumor viruses.

## 2. Polyomaviruses

The *Polyomaviridae* are small, non-enveloped, double-stranded DNA viruses that infect a wide range of species [33]. The family was named after the founding member, polyomavirus, which caused “many tumors” in mice [34], followed by the prototypic primate polyomavirus, Simian Vacuolating agent 40 (SV40), from the rhesus monkey [35]. The first two human polyomaviruses discovered in 1971, JC and BK, were named after the index cases, and cause serious disease in the immunocompromised [36]. The last decade has seen the discovery of several novel human polyomaviruses, including Merkel cell polyomavirus, which causes an aggressive skin cancer [37,38]. These discoveries have led to resurgence in interest in polyomavirus biology and to the roles of virus encoded proteins in pathogenesis. In this regard, two members of the family have recently been shown to encode proteins with viroporin characteristics.

### 2.1. JC Virus Agnoprotein

In 1958, progressive multifocal leukoencephalopathy (PML), a potentially fatal demyelinating disease of the brain, was discovered and later attributed to a novel polyomavirus termed JC [39]. JC is widespread amongst the adult population, with studies suggesting infection rates are upwards of 35%, and possibly as high as 90% [40]. Despite the high prevalence, PML incidence is extremely low due to the tendency of JC to result in an asymptomatic latent infection of the kidneys, lymphatic system and bone marrow in immunocompetent individuals [41]. Activation of the virus occurs almost exclusively in immunocompromised patients and is characterised by a lytic infection of oligodendrocytes resulting in demyelination and development of PML, although it is not clear when the virus infects the central nervous system. Before the introduction of antiviral therapies to combat the progression to AIDS, PML was a prominent feature of the HIV phenotype. Since then, there has been a resurgence in PML cases as a wide range of immunosuppressant therapies are being applied to combat autoimmune diseases such as multiple sclerosis (MS) and aid in the acceptance of transplanted tissues [42]. Links between JC and a range of human cancers have also been reported, although these are disputed [43].

Similar to most polyomaviruses, JC encodes for early proteins, which constitute the small and large T antigens and their many splice variants, which engage with many host processes to ensure a cellular milieu is available that is conducive to virus replication. In addition to this, three late structural proteins are expressed. These include the major capsid protein, VP1, and the VP2/VP3 minor capsid proteins [33]. JC encodes for an additional late protein termed agnoprotein [44]. Agnoprotein is only expressed by a limited number of polyomaviruses including the related BK and SV40.

The 71 amino acid agnoprotein is highly basic and contains a central hydrophobic region capable of forming an amphipathic helix [45,46]. In concert with residues in the amino terminal region, this amphipathic helix is required for localization to the ER and membrane insertion [11]. Biochemical analysis shows that residues 30-37 within the amphipathic helix are necessary and sufficient for dimer and oligomer formation [45]. Agnoprotein oligomers are stable in SDS and do not depend on disulphide bridge formation [45]. Recent nuclear magnetic resonance (NMR) data confirmed the formation of an amphipathic helix between Leu23 and Phe39 [46]. The basic nature of the protein may provide flexibility within the amino and carboxyl termini increasing the range of interactions with host partners. For a list of known JC agnoprotein binding partners see Table 1.

**Table 1:** Known JC agnoprotein binding partners [44]. Function and region of interaction are stated if known.

Host Encoded Interacting Partner	Function	Region of Agnoprotein Required for The Interaction
AP-3 (δ subunit)	Modulates vesicle trafficking, prevents agnoprotein degradation	Residues 1–12
PP2A	Dephosphorylation of JC agnoprotein	Residues 18–36
FEZ1	Facilitates virus release	Unknown
Tubulin	Unknown	Unknown
HP-1α	Nuclear egress of JC virions	N-terminus
p52	Unknown	Unknown
p53	Modulation of the cell cycle	N-terminus
p103	Unknown	Unknown
p112	Unknown	Unknown
p158	Unknown	Unknown
Ku70	Host cell DNA repair	N-terminus
YB-1	Altered host gene expression	Residues 18–36
**Virus encoded interacting partner**	**Function**	**Region of agnoprotein required for the interaction**
JC T-antigen	Repression of JC transcription and DNA replication	N-terminus
JC t-antigen	Disruption of the PP2A – t-antigen interaction	N-terminus
HIV-1 Tat	Inhibition of HIV1 gene expression	Residues 18–54

Viroporin activity is associated with increased plasma membrane permeability, resulting in elevated cytosolic calcium levels [11]. Similar to several known viroporins, agnoprotein expression manipulates host trafficking pathways to allow transport of agnoprotein to the cell surface to mediate plasma membrane permeabilization [47]. To achieve this, agnoprotein interacts with the δ sub-unit of adaptor protein complex 3 (AP-3), inhibiting its function and as a consequence preventing trafficking of agnoprotein to the lysosome for destruction. Substitution of two basic amino acids in the amino terminal region of agnoprotein (Arg8/Lys9) prevented the interaction with AP-3, perturbed sub-cellular localization and abrogated the plasma membrane permeabilization seen with the wild type protein. Moreover, JC viruses containing either an agnoprotein deletion or alanine substitution of the basic residues resulted in a comparable defect in virion release from infected cells [11,47]. The conclusion of these studies is that the basic residues are a critical requirement for agnoprotein function, potentially by contributing towards viroporin activity. Mutations of a similar basic motif in the HCV p7 protein also impaired viroporin activity, as assessed using a carboxylfluorescein dye release assay, however, further investigation in fact revealed that the mutant protein was no longer able to integrate correctly into membranes [10]. It is plausible that by mutating the basic loop in agnoprotein, membrane integration has been perturbed which would be expected to have broader impacts on agnoprotein function beyond inhibiting viroporin function.

Agnoprotein has multiple roles within the JC life cycle, and some of these are known to require residues within the amphipathic helix, and as such may depend upon viroporin function. Binding of the Large T antigen to the viral origin of replication is enhanced in the presence of agnoprotein [45]. Agnoproteins containing mutations that would be expected to impair amphipathic helix formation display decreased Large T binding and significantly reduced virus genome replication [45]. Some mutations within the amphipathic helix impact protein stability, resulting in reduced agnoprotein expression [46]. As such the impact on virus replication might arise as a result of less agnoprotein rather than loss of putative viroporin activity. Nevertheless, reduced JC replication is a feature observed in some agnoprotein deletion viruses, indicating that modulation of replication is a *bona fide* function of agnoprotein [48].

As polyomavirus virions assemble in the nucleus, they are first required to exit this organelle into the cytoplasm prior to the egress from the infected cell [49]. Since complete lysis of the nuclear membrane would abolish the integrity of the infected cell and impair virus replication, JC has evolved to instead alter the nuclear envelope (NE) to mediate virus egress. JC infected cells show protrusions and invaginations in the NE, mediated through binding of agnoprotein to heterochromatin protein-1 (HP-1) [50]. HP-1 normally interacts with the Lamin-B receptor (LBR) to assist with the reassembly of the NE after cell division [51]. However, the amino terminal domain of agnoprotein is capable of binding to HP-1 inducing disassociation from LBR and resulting in morphological changes to the NE, allowing escape of progeny virions [50]. Whether viroporin activity is required for this function is not clear, although binding to HP-1 requires the amino terminal 24 residues, which are outside the putative TMD.

Agnoprotein has been shown to be subject to extensive post-translation modification and this is likely a key regulator of function. Protein kinase C (PKC) can phosphorylate JC agnoprotein on three identified residues; Ser7, Ser11 and Thr21 [52]. JC viruses containing alanine substitutions at these sites were less able to maintain an active infection, although they displayed enhanced agnoprotein expression levels at early time points during infection. Subsequent studies identified an antagonistic role for protein phosphatase 2A (PP2A) in agnoprotein phosphorylation [53]. Agnoprotein dephosphorylated by PP2A was shown to inhibit JC replication to levels comparable with the alanine substitution mutants. Mechanistically, JC small T antigen has been shown to bind to both PP2A and agnoprotein, and is thought to reduce the ability of PP2A to dephosphorylate agnoprotein [53]. Interestingly, depletion of PP2A from JC infected cells using siRNA also resulted in reduced virus replication [53]. This raises the intriguing possibility that both phosphorylated and de-phosphorylated forms of agnoprotein have defined functions, and interplay between the two contributes to the successful replication and propagation of JC. These functions, however, have yet to be delineated. Phosphorylation has been shown to be important in regulating the sub-cellular localization of proteins, as such it is plausible that the phosphorylation state of agnoprotein may determine its sub-cellular location and hence the role it plays in replication. Whether it is also required to regulate viroporin function has not been shown. However, given the findings of Sawa and colleagues that viroporin function is needed at the cell surface, it is a possibility that phosphorylation of agnoprotein may also contribute to localizing the channel to where it is required [47]. Regulation of viroporins by post-translational modifications would add another tier of control to these important proteins.

**Figure 1:**
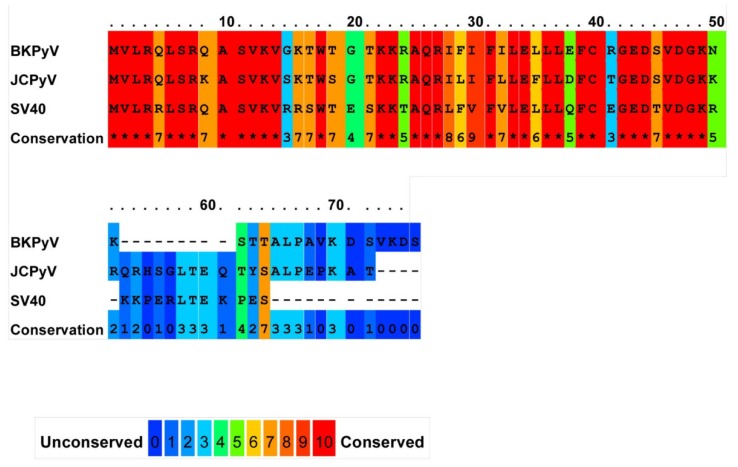
Sequence conservation of BK, JC and SV40 agnoproteins. Residues are displayed using amino acid single letter codes. Conserved residues are shown in red through to unconserved residues in blue. * represents a residue conserved across all three sequences. Uniprot sequences P03085 (BK), P03086 (JC) and P03084 (SV40) were used. The sequence alignment was generated using the online PRALINE multiple sequence alignment tool (http://www.ibi.vu.nl/programs/pralinewww/).

JC agnoprotein shares significant identity with the agnoproteins encoded by the related viruses BK and SV40 (60% across the whole protein, rising to 90% in the amino terminal region) [44] (Figure 1). As expected from such a high homology, agnoproteins of BK and SV40 appear to perform similar functions during the virus life cycle, although with some subtle differences. Though less well studied, loss of agnoprotein appears to correlate with defects in the late stages of the virus life cycle and to perturb egress. In addition, agnoprotein may also play a role in packaging the SV40 genome [48]. BK egress has recently been shown to be sensitive to the DIDS compound [54]. Given that DIDS is a broad-spectrum inhibitor of ion channels, it is possible that the target of its actions is an agnoprotein viroporin. It would be interesting to assess the impact of DIDS on virus egress from an agnoprotein knockout virus.

The wide-ranging roles of agnoproteins identified from both JC and BK coupled with the deleterious effects seen in the agnoprotein deficient viruses warrants a serious analysis of the potential for agnoprotein as a target of direct acting antiviral therapeutics. Groups have previously generated siRNA to target JC agnoprotein and have had success of inhibiting viral replication in mice infected brains, justifying the premise of targeting this protein for inhibition [55]. Such studies would be expedited by a direct analysis of channel activity using recombinant agnoprotein, similar to the pioneering work on the HCV p7 protein [56]. Not only would this affirm channel function but would also provide a platform from which to screen the large libraries of compounds displaying viroporin inhibitory properties available.

### 2.2. SV40 Late Proteins

Three late proteins have been shown to aid in SV40 entry and release by virtue of their viroporin-like properties. VP2 and VP3 are generated from successive Met residues within the VP2 messenger RNA so they share a common carboxyl-terminus. They are classed as minor constituents of the virus particle, being present at a stoichiometry of one copy of a VP2 or VP3 protein per VP1 pentamer [57]. Given that SV40 is a non-enveloped virus, it has evolved strategies to allow transport of the incoming virion through the membranous environment of the cytoplasm to the nucleus to initiate genome replication. A number of studies have shown that both VP2 and VP3 form pores in cellular membranes and may aid in delivery of the SV40 virion into the nucleus [58,59]. Mutation of residues within putative TMD prevented membrane targeting and when engineered into SV40 genomes resulted in reduced infectivity [60].

SV40 also encodes a unique very late protein termed VP4, encoded by the same transcript as VP2/VP3. Unlike other late proteins, VP4 is thought not to be incorporated into SV40 capsids. VP4 encodes a single TMD and associates with membranes, where it generates a channel of defined pore size and increases membrane permeability. VP4 is expressed at least 24 h later than other late proteins, indicating a potential role in virus release [61]. VP4 channel activity is regulated by the lipid environment and shows a greater activity in liposomes mimicking the composition of the plasma membrane, and SV40 viruses lacking VP4 exhibit a significant defect in virus spread. Together, these data indicate that VP4 is a viroporin that functions specifically during the latter stages of the SV40 life cycle [61,62,63,64]. Whether other mammalian polyomaviruses encode a VP4 protein is not clear. It is currently uncertain why SV40 might require VP4 when other polyomaviruses do not. JC agnoprotein has been shown to permeabilize the plasma membrane [11], and may therefore fulfil this role. However, given the sequence similarity it is likely that SV40 agnoprotein may also serve as a viroporin. It is possible that there is either redundancy or co-operation between agnoprotein and VP4. These questions will remain unanswered until viroporin function is confirmed in SV40 agnoprotein.

## 3. Papillomaviruses

The *Papillomaviridae* contains an extensive array of different papillomavirus (PV) types capable of infecting a variety of animal species, of which at least 170 have currently been isolated from humans [65]. Like Polyomavirus, they are small, non-enveloped double stranded DNA viruses around 55 nm in diameter [66]. Most PV encode six early (E1, E2, E4, E5, E6 and E7) and two late structural genes (L1 and L2). Approximately 12 HPV types, termed high-risk, are the causative agents of several ano-genital and oral malignancies [67]. Of these, HPV16 and HPV18 are the most important and are responsible for approximately 70% of the cervical cancer cases, and for the deaths of approximately 270,000 women in 2012 alone [66]. Low risk HPV are usually cleared by the body and vaccines are available for prophylactic treatment of high risk HPV, but there is as of yet no therapeutics for existing cases of HPV infection.

### HPV16 E5

**Figure 2:**
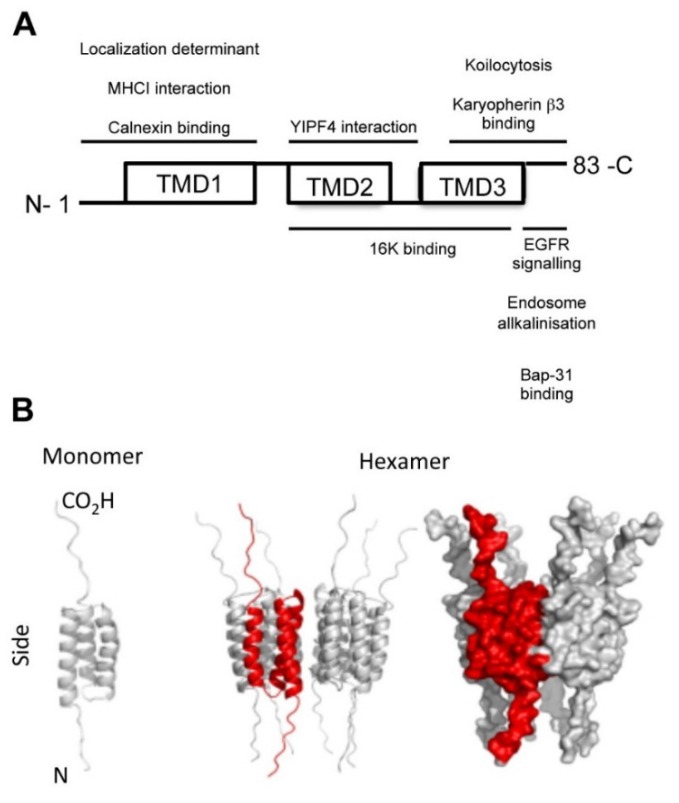
HPV16 E5 is a 3 TMD viroporin. (**A**) HPV16 E5 is predicted to have three transmembrane domains (TMDs; boxes) based on the hydrophobic nature of its amino acids. Membrane permeabilization assays demonstrate that the carboxyl-terminus extends into the cytosol while the amino-terminus is directed towards the endoplasmic reticulum (ER) lumen. The first TMD gives rise to the subcellular localization of E5 and mediates binding to MHC class I molecules and Calnexin. The second TMD facilitates the recently identified interaction with the transmembrane protein YIPF4 as well as the 16K subunit of the H^+^-ATPase. Further functions of E5 such as the increase of koilocytosis, activation of EGFR signaling and induction of endosome alkalinization are exerted via the third TMD; (**B**) The sequence of HPV16 E5 was obtained from Uniprot and the secondary structure predicted using PSIPRED and MEMSAT-3 and energy minimized. The model for an E5 monomer contained three TMD and had the lowest energy and was used to build a hexameric model using the protocol described previously [81]. Each monomer in the model was minimized individually to restore the symmetry and was refined using prime^2^ (Schrodinger Inc). The oligomeric state of HPV16 E5 was confirmed by native PAGE and transmission electron microscopy [3].

Three gene products E5, E6 and E7 mediate the transforming potential of high-risk HPV. E6 and E7 are the major drivers of keratinocyte proliferation [68]. They are necessary to maintain the cell cycle of differentiating keratinocytes and they achieve this by binding to and inactivating the Retinoblastoma (pRb) and p53 tumor suppressor proteins [68]. Repression of E6 and E7 in various cell lines has been shown to induce cellular senescence and halt differentiation. The E5 protein is the least understood of the three oncoproteins [69]. HPV16 E5 is a highly hydrophobic, 83 amino acid membrane protein that resides in the lumen of cytoplasmic membranes and interacts with a growing number of cellular partners [3,69,70,71,72,73] (Figure 2A). The triple membrane spanning topology of HPV16 E5 was determined by partial membrane permeabilization fluorescence studies in cells and these support the idea that the E5 amino terminus resides in the lumen of the ER with a carboxyl terminus exposed to the cytosol [74]. Lack of specific antibodies preclude localization studies from virus infected cells, however, epitope tagged E5 has been shown by over-expression studies to predominantly localize to ER and Golgi membranes [73]. Cell surface localization has also been noted [75], although this has been disputed by others [74]. Sequence analysis demonstrates that a recognizable E5 gene is missing from several HPV types (beta, gamma and mu). Further, viruses that do encode an E5 open reading frame show significant sequence divergence, with the resulting protein product ranging in size from approximately 40–90 amino acids. Such significant divergence might indicate distinctive roles for E5 within the HPV life cycle, dependent on the specific virus type. Importantly, all high-risk cancer causing HPV types encode an E5 protein similar to HPV16, of approximately 80 acids termed E5 alpha [76]. HPV16 E5 oligomerizes *in vitro* and in cells, with oligomer formation driven not by the presence of disulphide linkages between cysteine residues, rather by hydrophobic interactions between individual monomers [3,77]. In 2012 our laboratory demonstrated that the E5 oligomer could mediate the controlled release of the small molecule carboxyflourescein using a liposome dye release assay [3]. Whilst some questions have been raised as to the validity of this indirect measure of channel activity [78], it has been widely used with multiple channels (e.g., HCV p7, classical swine fever virus (CSFV) p7, respiratory syncytial virus (RSV) SH), and has provided important insights into their activity [31,79,80]. The stoichiometry of HPV16 E5 was predicted to be hexameric using *in silico* modelling and subsequently confirmed by native PAGE electrophoresis and transmission electron microscopy [3] (Figure 2B). These complexes displayed channel-forming activity with a defined pore size, and activity was increased in acidic pH. Sensitivity of E5 to the adamantane derivative Rimantadine [3] and the alkyl imino-sugar *N*N-DNJ (our unpublished data) was demonstrated *in vitro*, as well as to a novel small molecule inhibitor generated using *in silico* modelling of the E5 channel and subsequent docking analysis [3].

E5 expression induces anchorage-independent growth in murine NIH3T3 cells and mitogenic effects in primary human foreskin epithelial cells [82,83]. Transformation has been recapitulated *in vivo* using transgenic mouse models where E5 is expressed in epithelial cells under the control of the Keratin-14 promoter. In these mice, E5 expression was associated with hyperplasia and tumor formation [84,85]. A number of studies have demonstrated the importance of the epidermal growth factor (EGF) receptor (EGFR) for E5-induced transformation [86,87], and these have since been reinforced by genetic studies which showed that transgenic mice expressing E5 failed to produce tumors when crossed with mice encoding for an inactive EGFR [85]. Thus the contribution of E5 to host cell transformation appears dependent on manipulation of the EGFR. Studies aimed at understanding the role of E5 in the productive HPV life cycle show that E5 is required to maintain the proliferative status of infected cells as they undergo terminal differentiation, in order to allow virus genome replication. Moreover, E5 causes a delay to the early stages of keratinocyte differentiation to achieve this. Using small molecule inhibitors targeting EGFR we have been able to show that these processes are also dependent on active EGFR signalling (our unpublished data). The precise mechanism by which E5 manipulates the EGFR is unclear. The current consensus model is that E5 expression is associated with a deacidification of endosomes [88]. This prevents the normal degradative trafficking pathways, which would culminate in deposition of active EGFR in the lysosome and termination of EGFR signalling. In E5 expressing cells active EGFR appears to be re-routed into recycling endosomes and returned to the plasma membrane, where it maintains mitogenic signalling [87]. Deacidification of endosomes might result from specific interactions with host binding partners [89], or, similar to IAV M2 and hepatitis C virus p7, might indicate a requirement for direct proton channel activity [10,17]. In support of this idea, enhanced EGFR signalling observed in E5 expressing cells was reduced by the small molecule inhibitors that prevented carboxyfluorescein release in the *in vitro* dye release assays [3]. These data suggest that the oncogenic activity of E5 may be directly linked to viroporin function. If this proves to be the case then E5 would represent the first example of an oncogenic viroporin. The development of specific mutant E5 proteins that lack channel activity will need to be tested in these assays in order to validate the small molecule inhibitor studies, which can be fraught with off-target effects. Moreover, it will be of great interest to generate viruses containing these mutants in order to study at what stage of the HPV life cycle any putative viroporin function is necessary. The viroporin function was described for E5 from HPV16 [5]. Given that E5 proteins from the other high-risk HPV types are predicted to adopt a similar three TMD topology, it will be of interest to determine whether viroporin function is conserved amongst this group of viruses. Whilst the presence of a defined pore size of <2 nm was confirmed in these studies [5], future work should focus on determining the ion selectivity of the E5 channel to conclusively determine whether there is a preference for protons or whether ion selectivity fluctuates dependent on the particular membrane environment, as has been shown for other viroporins [90]. Finally, although E5 is not thought to be expressed in cervical cancer cells due to the integrated nature of the genome [69], it is postulated to play a role in the early stages of cancer development. Given the limited treatment options for those already infected with HPV, ongoing research into the development of E5 inhibitors with greater potency suited to drug development programs appears feasible given our findings [3]. Thus, there is potential for drug development targeting E5, yet whether this will ultimately prove relevant in the post-vaccination era remains to be seen.

## 4. Concluding Remarks

Viroporins are multi-faceted viral proteins shown to play key roles in the life cycles of a number of important human pathogens. The relatively recent identification of such proteins encoded by small DNA tumor viruses offers the opportunity to understand their contribution towards the productive life cycle of these viruses and ultimately their pathogenesis. Analysis of viroporin deletion mutants in these viruses will undoubtedly help to delineate their functions and may provide an important insight into how they manipulate critical host functions. The large economic and health burden associated with virus infection, alongside the rapid increase in resistance to existing therapeutic regimes, if available, demonstrates the need for new anti-viral drugs. Given the low mutation rate of DNA virus genomes, identifying virus encoded targets as exemplified by the viroporins presents an attractive target for future therapeutics. Increasing understanding of viroporin structure and function, along with the advent of high throughput technologies will hopefully lead to the development of these much needed treatments.
